# A Rare Case of Hyperhomocysteinemia-associated Thrombotic Stroke in the Pediatric Age Group

**DOI:** 10.7759/cureus.4490

**Published:** 2019-04-17

**Authors:** Hitanshu Dave, Parth Dalal, Pooja Patel, Rupak Desai

**Affiliations:** 1 Internal Medicine, Hackensack Meridian Health Jersey Shore University Medical Center, Neptune City, USA; 2 Pediatrics, NKP Salve Institute of Medical Sciences and Research Center, Nagpur, IND; 3 Rheumatology, Advocate Aurora Healthcare, Brookfield, USA; 4 Cardiology, Atlanta Veterans Affairs Medical Center, Decatur, USA

**Keywords:** stroke, thrombotic stroke, homocysteine, pediatric, tenth cranial nerve, hyperhomocysteinemia, vertigo, young patient, syncope, ninth cranial nerve

## Abstract

Association between hyperhomocysteinemia and stroke has been well documented in the literature. However, there are limited reports revealing stroke events in the pediatric population affected by hyperhomocysteinemia. Herein, we present a case which shows genetically inherited hyperhomocysteinemia leading to a stroke event in a 14-year-old child. The patient presented to the outpatient clinic with dizziness and nonprojectile vomiting since the previous day without any history of weakness in the extremities or unconsciousness. The preliminary neurologic examination showed positive right-sided cerebellar signs like ataxia on physical examination and the final diagnostic workup confirmed acute non-hemorrhagic bilateral cerebellar and medullary infarction with hyperhomocysteinemia. We discuss the case presentation, diagnostic workup, and management strategies.

## Introduction

It is being hypothesized that homocysteine is highly reactive amino acid and potentiates oxidation of very low-density lipoprotein which is toxic to endothelial cells and can cause arterial and venous thrombosis [1]. Consistently, the association between hyperhomocysteinemia and stroke has been well documented in the literature [2]. However, there are limited reports revealing stroke events in the pediatric population affected by hyperhomocysteinaemia. Herein, we present a case which shows genetically inherited hyperhomocysteinaemia leading to a stroke event in a child. A 14-year-old male child presented to the outpatient clinic with dizziness and nonprojectile vomiting for one day without any history of weakness in the extremities or unconsciousness. The preliminary neurologic examination showed positive right-sided cerebellar signs like ataxia on physical examination. He had a similar history in the past after a fall and a positive history of similar complaints in his father. We lay out the detailed case presentation, diagnostic workup of acute non-hemorrhagic bilateral cerebellar and medullary infarction with hyperhomocysteinaemia and management strategies we employed.

## Case presentation

A 14-year-old male patient presented to the outpatient clinic with dizziness since the previous day without any history of loss of consciousness, weakness in the extremities or seizure episode. He also complained of non-bloody, nonbilious, and projectile vomiting with a negative history of abdominal pain and diarrhea. In the past, he had identical complaints of dizziness one and half months back after falling off his bicycle. His parents had a nonconsanguineous marriage. There was a positive family history with his father suffering premature cardiovascular death at the age of 35 years.

On presentation to the clinic, he was in a hemodynamically stable state. Neurological evaluation was normal without any complaints of weakness, positive Babinski sign, or sensory involvement. He was admitted on the floor for thorough evaluation for his vertigo. Local causes of vertigo were ruled out on initial evaluation by an ear, nose, and throat consultation. Ophthalmology evaluation was done to rule out causes of raised intracranial tension, which showed no evidence of papilloedema on indirect ophthalmoscopy. The patient was reviewed anthropometrically which showed an increased arm length more than height.

The patient suddenly became drowsy along with complaints of right-sided weakness and continuous hiccups with high fever spikes, thus he was shifted to pediatric intensive care unit and his neurological assessment showed upper motor neuron facial nerve palsy as evident from right-sided hemiparesis.

The magnetic resonance imaging showed bilateral cerebellar non-hemorrhagic infarcts. Later, the patient deteriorated and developed respiratory distress along with pooling of secretions, hoarseness of voice, and deviation of uvula to the right side with an absent gag reflex, thus, suggesting a medullary component with ninth and tenth nerve involvement.

Other examinations including complete blood count, coagulation profile, 2D (transthoracic) echocardiogram, and electrocardiogram were found to be normal and helped us to rule out arrhythmias for syncope workup. The antinuclear antibody assessment was negative and helped to rule out any autoimmune disorder. Magnetic resonance angiography showed a vascular loop of the anterior inferior cerebellar artery around the seventh cranial nerve on the right side. Furthermore, he had elevated homocysteine levels (18.54 micromoles/liter). Thus, he was diagnosed with acute non-hemorrhagic bilateral cerebellar and medullary infarction with homocysteinemia after a careful assessment and exclusion of all relevant differential diagnoses.

The patient was given supportive treatment while admitted to the pediatric intensive care unit. He was treated by oral antispasmodics for spasms and anti-emetics for vomiting. For homocysteinemia, he was managed with oral pyridoxine, folic acid, and aspirin. On follow-up visits, the patient was doing well.

## Discussion

Elevated levels of homocysteine (>15 micromoles/liter) is considered a major risk factor for non-hemorrhagic stroke [2]. Homocysteine gets eliminated from the body through vitamin B12 metabolism and folate metabolism finally converting into adenosine which gets cleared from the body. Thus, a deficiency of folate or vitamin B12 can lead to an increased level of homocysteine [3].

The etiologies of hyperhomocysteinaemia are multifactorial. Non-genetic determinant etiology includes dietary deficiency whereas genetic determinants include enzyme defects such as classical homocystinuria due to deficiency of cystathionine beta-synthase. It can also occur due to a defect in methylcobalamin formation leading to the triad of megaloblastic anemia, homocystinuria, and hypoketonemia [3].

This case highlights the fact that when a young patient presents with an incident stroke event along with other supportive systemic findings with or without a family history, homocysteinemia should form a major differential diagnosis to expedite the diagnostic screening and further timely management strategies [4-6]. Table 1 displays the differential diagnoses considered for this case and the causes for exclusions. Young patients presenting with vertigo with associated neurological signs should be suspected of cerebellar stroke along with other causes of stroke such as thrombotic disorders such as protein C deficiency and factor V Leiden mutations should be ruled out. This patient did not present with cerebellar signs but during the course of the stay, he developed cerebellar signs. Therefore, a young patient with symptoms like syncope or vertigo should undergo a complete neurological assessment both clinically as well as by imaging. They should also be evaluated metabolically as they can also present with stroke-like representation [4]. Although young-onset strokes more common in developed nations as compared to developing nations, it is imperative to rule out a history of substance abuse and rare etiologies like moyamoya disease as that can lead to acute cardiovascular and cerebrovascular events in young individuals [7-10].

**Table 1:** Differential diagnosis ruled out for the case.

Differential Diagnosis	Reasons to Rule Out
Folic acid deficiency	Normal folic acid levels
Vitamin B12 deficiency	Vitamin B12 level lab test for exclusion, absent neurological symptoms.
Arterial-venous malformation	Negative magnetic resonance imaging scan and mainly causes hemorrhagic stroke
Protein C deficiency	Normal protein C levels can present with multiple blood clots in the body.
Antithrombin – 3 deficiency	Antithrombin levels normal.
Congenital heart disease	Normal 2D (transthoracic) echocardiogram to rule out atrial septal defect and ventricular septal defect.

## Conclusions

A young pediatric patient with stroke-like features should be immediately evaluated for thrombotic disorders like protein C deficiency and hyperhomocysteinaemia in addition to any metabolic derangements. Cerebellar infarcts typically present with complaints of vertigo in this age group.
